# Phylogeography using mitogenomes: A rare Dipodidae, *Sicista betulina*, in North‐western Europe

**DOI:** 10.1002/ece3.8865

**Published:** 2022-04-21

**Authors:** Liselotte Wesley Andersen, Magnus W. Jacobsen, Jane Frydenberg, Julie Dahl Møller, Thomas Secher Jensen

**Affiliations:** ^1^ Department of Ecoscience Aarhus University Aarhus C Denmark; ^2^ Section for Marine Living Resources National Institute of Aquatic Resources Technical University of Denmark Silkeborg Denmark; ^3^ Højbjerg Denmark; ^4^ Møller Consultansy Frøstrup Denmark; ^5^ Natural History Museum, Aarhus Aarhus Denmark

**Keywords:** divergence time, genetic diversity, mitogenomes, Phylogeography, population structure, *Sicista betulina*

## Abstract

Repeated climatic and vegetation changes during the Pleistocene have shaped biodiversity in Northern Europe including Denmark. The Northern Birch Mouse (*Sicista betulina*) was one of the first small rodent species to colonize Denmark after the Late Glacial Maximum. This study analyses complete mitochondrial genomes and two nuclear genes of the Northern Birch Mouse to investigate the phylogeographical pattern in North‐western Europe and test whether the species colonized Denmark through several colonization events. The latter was prompt by (i) the present‐day distinct northern and southern Danish distribution and (ii) the subfossil record of Northern Birch Mouse, supporting early Weichselian colonization. Samples from Denmark, Norway, Sweden, Russia, Latvia, Estonia, and Slovakia were included. Mitogenomes were obtained from 54 individuals, all representing unique mitogenomes supporting high genetic variation. Bayesian phylogenetic analysis identified two distinct evolutionary linages in Northern Europe diverging within the Elster glaciation period. The results of the two nuclear genomes showed lower genetic differentiation but supported the same evolutionary history. This suggests an allopatric origin of the clades followed by secondary contact. Individuals from southern Denmark were only found in one clade, while individuals from other areas, including northern Denmark, were represented in both clades. Nevertheless, we found no evidence for repeated colonization's explaining the observed fragmented distribution of the species today. The results indicated that the mitogenome pattern of the Northern Birch Mouse population in southern Denmark was either (i) due to the population being founded from northern Denmark, (ii) a result of climatic and anthropogenic effects reducing population size increasing genetic drift or (iii) caused by sampling bias.

## Graphical abstract

The phylogeographical pattern of the Northern Birch Mouse (*Sicista betulina*) in Northwestern Europe was investigated using complete mitochondrial genomes and two nuclear genes. Samples representing Denmark, Norway, Sweden, Russia, Latvia, Estonia and Slovakia were analysed. Mitogenomes were obtained from 54 individuals, all unique, supporting high genetic variation. Two distinct evolutionary linages diverging within the Elster glaciation period was found suggesting an allopatric origin and vicariance followed by secondary contact throughout the northern range. The results indicated that the mitogenome pattern of the Northern Birch Mouse population in southern Denmark was either (i) due to the population being founded from northern Denmark, (ii) a result of climatic and anthropogenic effects reducing population size increasing genetic drift or (iii) caused by sampling bias.

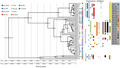

## 1 INTRODUCTION

The present species pool and biogeography of European plant and animal populations are strongly shaped by repeated climate changes during the Pleistocene epoch and by farming practices during the mid‐ and late Holocene (Emanuelsson, 2009; Lang, 1994). These climate changes have impoverished the rich Miocene and Pliocene European flora (Mai, 1995; Svenning, 2003) and over much of the northern Europe extensive Pleistocene ice sheets (Ehlers & Gibbard, 2004) have resulted in recurrent “tabula rasa” situations.

**FIGURE 1:**
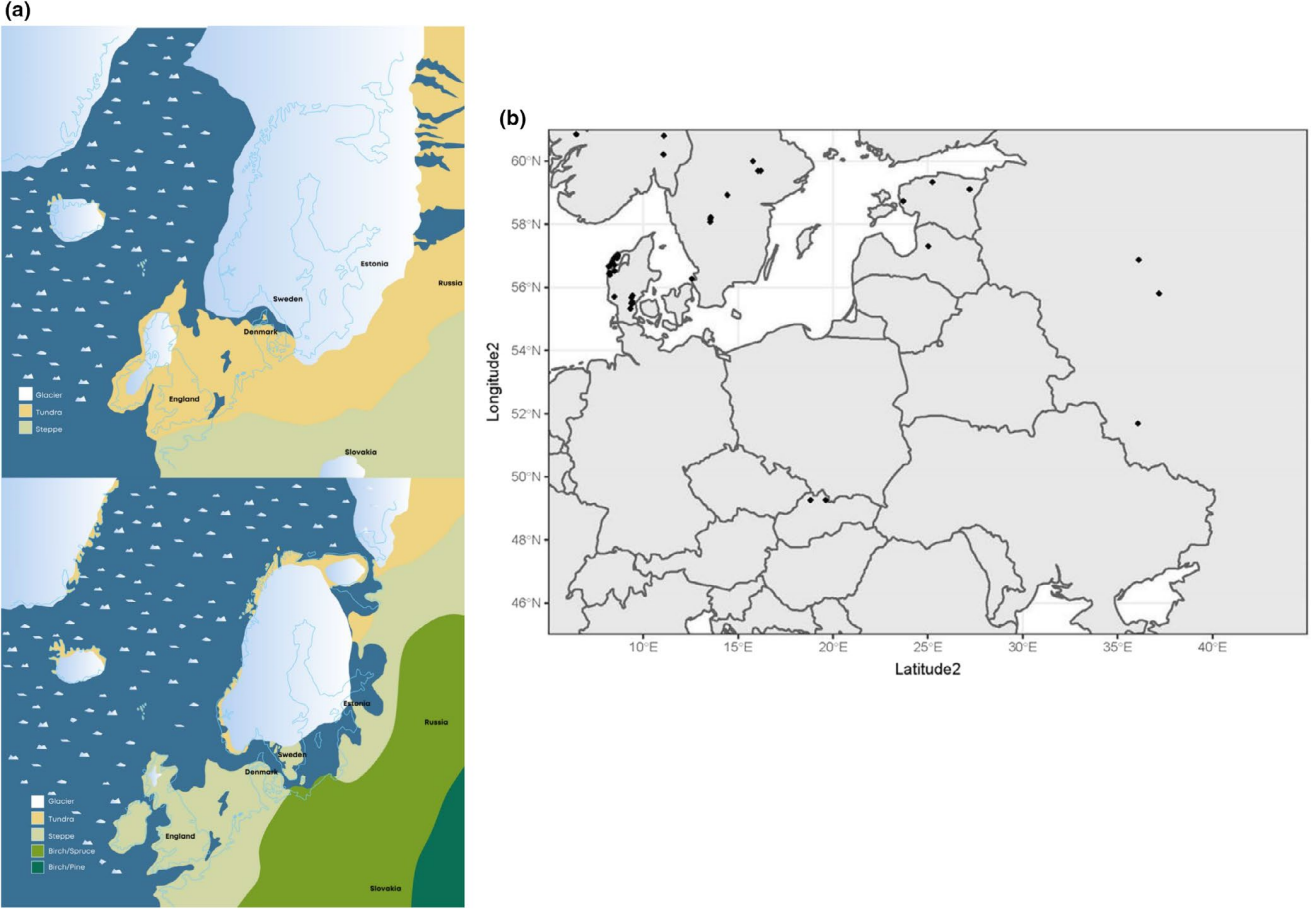
(a) North‐western Europe about 16,000 years ago (above) and North‐western Europe about 14,000 years ago (below; Aaris‐Sørensen, 2007). (b) the sampling locations of the Northern Birch Mouse analyzed in the study

Until the Latest Glacial Maximum (LGM) around 20,000 years ago, northern Europe including parts of Denmark was covered by ice. During the Late‐ and Post‐glacial periods when the glaciers retreated, a succession of habitat types from tundra to deciduous and coniferous forests occurred. Immigration waves of colonizing fauna started as early as in the last part of the de‐glaciation stage (c. 17,000–15,000 years ago; Figure 1a). In the beginning, immigration of faunas from east and south was facilitated by a continuous continent; however, due to rising sea water levels and lowered landmasses, around 8000 years ago, continental Denmark was transformed into a group of islands and one peninsula (Aaris‐Sørensen, 2016), creating barriers to further immigration leading to local extinctions. Britain became an island, as the land between Denmark and Britain (Doggerland) was flooded, and a strait between Denmark and southern Sweden, The Sound, separated these two countries.

The colonization pattern of flora and fauna after LGM was mainly influenced by changes linked to these climatic changes during the late Quaternary (Hewitt, 2004), and the expansions and contractions in the distribution of flora and fauna caused demographic, and spatial events that influenced the pattern of genetic diversity (Hewitt, 2004).

The post‐LGM expansion in Europe described by Hewitt (1999) started from southern and eastern refugia with plants and animals moving northwards following a series of rapid temperature increases. However, Pedreschi et al. (2019) suggested that individual species reacted more niche‐specific to climate oscillations, thus questioning the generalization of this pattern across species by implying the existence of additional refugia further north or west in Europe depending on the species in question, that is *Microtus arvalis* (Pedreschi et al., 2019) and *Clethrionomys glareolus* (Marková et al., 2020). Nevertheless, in most species, genetic diversity is generally higher in populations in the southern areas, as compared with the more recently colonized northern regions. This pattern follows the central‐marginal hypothesis predicting a decrease in genetic diversity with distance from the glacial refugium (Eckert et al., 2008) and is supported by many phylogeographical studies of animal and plant species.

Until recently, phylogeographic studies primarily relied on the analysis of short fragments or single genes from the mitochondrial genome (mitogenome). Analyzing the full mitogenome (ca. 16–18,000 bp long (Ballard & Whitlock, 2004)) increases the genetic resolution, which is especially important when analyzing closely related populations where diagnostic variation may be limited (Jacobsen et al., 2012; Morin et al., 2010). Most studies have assumed that variation evolves through neutral processes, as the mitogenome is prone to genetic drift given its small effective size (*N*_e_; approximately four times smaller than its nuclear counterparts), due to a haploid nature and maternal inheritance (Ballard & Whitlock, 2004; Birky et al., 1983; Galtier et al., 2009). Genetic drift in combination with strong purifying selection are the two predominant evolutionary forces within the mitogenome. Nevertheless, positive selection might still act on individual genes or single codon positions (Ballard & Whitlock, 2004; Galtier et al., 2009). Several studies have shown evidence for positive selection in a wide array of taxa such as fish and mammals (e.g., Belanger‐Deschenes et al., 2013; da Fonseca et al., 2008; Fontanillas et al., 2005; Gagnaire et al., 2012; Garvin et al., 2011; Jacobsen et al., 2014, 2015). Such changes may be linked to metabolic differences and ultimately cover adaptations that are vital for the survival of the specific populations or species. Positive selection may also influence divergence between populations and hence impede reliable estimation of divergence time.

This study focuses on the phylogeographic pattern and colonization history of the Northern Birch Mouse (*Sicista betulina*), (Dipodidae: Zapodinae). This species was among the first rodent immigrants to arrive in Northern Europe and subfossil remains from Denmark date to the Late Glacial period, ~14,000 years ago, when the land was covered by a tundra‐boreal forest‐like vegetation (Aaris‐Sørensen, 1995). The *Sicista* genus shows highly variable karyotypes and cluster into five major species groups: *betulina*, *caucasica*, *caudate*, *tianschanica*, and *concolor* (Lebedev, Poplavskaya, et al., 2020). The *betulina* group is divided into a Northern species group consisting of *S*. *betulina* and Strand's Birch Mouse (*S.* *strandi*) and a Steppe group consisting of amongst others, the Southern Birch Mouse (*S*. *subtilis*; Holden et al., 2017; Lebedev, Rusin, et al., 2019). Present‐day range of Northern Birch Mouse shows a very fragmented distribution in its western part, including Denmark, Norway, Germany, Austria, Czech Republic, Slovakia, and Sweden, and a more continuous distribution from Poland, Finland, and the Baltic states further east into Russia until the Baikal Lake area. Strand's Birch Mouse is distributed in the southern part of Russia, while *S*. *subtilis* is distributed in southern Russia from the river Don eastwards (Supplementary S1; Cserkész & Kennerley, 2017; Kovalskaya et al., 2011; Lebedev, Rusin, et al., 2019).

The origin of the genus *Sicista* can be referred to Asia (Kimura, 2011), but in Europe, fossil remains in southeastern France suggest that *S*. *betulina* probably appeared during Middle Pleistocene, around 781,000 to 126,000 years ago (Rofes et al., 2012). Further fossil finds of *S*. *betulina* are reported from Jersey—La Cotte de St. Brelade from around Middle‐ to Late Pleistocene in the Saalian and Weichselian layers (380,000–11,500 years ago; Chaline & Brochet, 1986) and from Boxgrove, West Sussex dating ~Middle Pleistocene (around 550,000–300,000 years ago; AHOB database—Pettitt & White, 2012). These findings suggest that the Northern Birch Mouse may have been present in Northern Europe during interglacial periods, with its range being retracted during the Weichselian glaciation to refugia further south and east within the tundra‐forest habitats. Fossil records are found in northern Denmark—in the Nørre Lyngby excavation—that can be dated to ~11,500 BC (Aaris‐Sørensen, 1995). In Germany and Hungary, fossil records are dated to ~Middle Pleistocene (around 781,000 to 126,000 years ago) supporting the finds from Jersey and West Sussex, and in Austria, Poland, Switzerland, Romania, Italy, and Spain, the oldest fossils are recorded from Late Pleistocene (126,000–11,430 years ago; Rofes et al., 2012). Given the presumably long persistence of Northern Birch Mouse in Europe, it is likely that several refugia existed during the latest ice age, as observed for many other mammals (Hewitt, 1999, 2004).

The climatic oscillations following the LGM which led to several glacial comebacks might have prompted several colonization waves of the Northern Birch Mouse into Northern Europe, including Denmark. These may have represented expansions from different refugia with different evolutionary histories, which might explain the observed highly fragmented distribution of the species today. If so, this is expected to be reflected in the genetic and phylogeographic patterns of the populations.

Here, we investigated the phylogeographical patterns of the Northern Birch Mouse in Northern Europe with special focus on Denmark. To do this, we analyzed full mitochondrial genome sequences (mitogenomes), in combination with genetic variation found in two nuclear genes: beta‐myosin heavy‐chain (MYH6) and the entire intron 7 of β‐fibrinogen (FGB). The samples covered two areas in Denmark (north and south), Norway, Sweden, Estonia, Latvia, Russia, and Slovakia and included an individual of Strands Birch Mouse (*S*. *strandi*), a sister species to the Northern Birch Mouse (Cserkész et al., 2015). The colonization history was investigated analyzing the phylogenetic relationship between the mitogenomes and nuclear haplotypes amongst the different sampling locations, estimating divergence time between major phylogeographical clades and populations. Selection was further analyzed to test for deviations from neutrality, and to assess whether adaptive variation existed between populations and species. For the Danish Birch Mouse, which are separated into a northern and southern population (Jensen & Møller, 2007), we tested whether the present populations represented one or more refugia by estimating divergence time using IMa software (Hey & Nielsen, 2007), and examining the population structure. Specifically, we tested whether the population of southern Denmark was founded by individuals originating from a different refugium compared with the population of northern Denmark. Alternatively, individuals from southern and northern Denmark may have originated from the same refugium and colonized Denmark concurrently. This would imply that the current fragmentation results from a mixture of climatic changes and anthropogenic activities that have prompt modifications in the landscape vegetation.

## 2 MATERIALS

DNA analyses were based on samples from museum collections (Swedish Museum of Natural History, Hungarian Natural History Museum, Natural History Museum of Denmark, Natural History Museum, Aarhus, Denmark) and collections from institutions (Institute of Vertebrate Biology, Czech Republic, Institute of Ecology and Evolution, Russia, Nature Conservation Agency, Latvia) and individual persons (Jeroen van der Kooij, Norway, Julie Dahl Møller, Denmark). Most samples were taken from frozen material, some from material preserved in alcohol. Altogether, 69 specimens were analyzed. One sample of Strand's Birch Mouse was included.

## 3 METHODS

### 3.1 Molecular methods

DNA was extracted from 69 specimens representing eight different European locations (Figure 1b) using a modified CTAB method (Andersen et al., 2004). Complete mtDNA sequences were obtained from 53 *S*. *betulina* and one specimen of *S*. *strandi*, which was used as an outgroup. Additional two nuclear genes, beta‐myosin heavy‐chain (MYH6 (241 bp), *N* = 66) and the entire intron 7 of β‐fibrinogen (FGB (591 bp), *N* = 69), were sequenced to increase the number of unlinked genetic markers (Matocq et al., 2007). Three individuals failed to amplify in MYH6. These were DK_3S, DK_11N, and DK_34N. FBG and MYH6 was chosen as Matocq et al. (2007) detected 114 and 15 informative sites/characters, respectively, in a sequence of 573 bp (FBG) and 238 bp (MYH6) in the woodrat genus Neotoma (Rodentia: Muridae). Consequently, we expected to find genetic variation in these nuclear genes in the Northern Birch Mouse as well. The species‐specific primers for the mtDNA were developed from other Murinae (*Microtus rossiaemeridionalis* (DQ015676.1), *Apodemus agrarius* (HM034866.1) Triant & Dewoody, 2006) using complete mitochondrial genome sequences downloaded from GenBank as baseline sequences (Supplementary S2). This was conducted by an iterative process, that is Sanger‐sequencing and re‐designing primers. To avoid numts, the complete mitochondrial genome was amplified using three long fragments of more than ~3 kb (Richly & Leister, 2004). The PCRs were conducted in a total volume of 20 µl using GOTaq Long PCR master mix (Promega) using the thermal profile: 95°C for 15 min, 40 cycles of 94°C for 45 s, ann. 48°C (primer3+6) for 45 s, extension of 72°C for 7 min, and finally extension of 72°C for 10 min. Annealing temperature for primer 2 + 9 was 55°C and identical extension time. For primer 7 + 8, annealing temperature was 66°C and extension time was 72°C for 3 min. The nuclear genes were amplified according to Matocq et al. (2007) and Sanger‐sequenced at MACROGEN, Europe. PCR fragments for mitogenomic analysis were cleaned and concentrations measured using a Qubit 2.0 Flourometer. Libraries were subsequently prepared using NEBNext^®^ Fast DNA Fragmentation & Library Prep Set for Ion Torrent following the manufacturer's recommendations. Libraries were tagged using Ion express barcodes (1–24) for multiplexing and sequencing was conducted using Ion express template 200 chemistry (paired end technology) on 314 and 316 chips according to the manufacturer's protocol. After assembly, not all mitogenome sequences were complete; hence, primers were designed using the mitogenomes as template filling the gaps using Sanger‐sequencing (MACROGENE Europe).

### 3.2 Mitogenome assembly and annotation

Sequences were assembled using Sequencher^®^ 5.2.3 (Gene Codes Cooperation) and complete mitochondrion sequence from *Microtus rossiaemeridionalis* as baseline sequence to help de novo assembly of the sequences in the first step. Reads with a coverage ranging from 10 to 300 were used as the total number of reads from each individual varied. The assembly was conducted choosing the longest contigs with the highest coverage. The generated mitogenome sequences were aligned to the annotated mitogenome sequence of *Apodemus agrarius* (Accession no. JN629047) in GENEIOUS 7.1.4 (Kearse et al., 2012) identifying the 13 genes in mtDNA (NAD1, NAD2, COX1, COX2, ATP8, ATP6, COX3, NAD3, NAD4|, NAD4, NAD5, NAD6, and COB) and D‐loop.

### 3.3 Nuclear genes and haplotype estimation

To identify the haplotypes of the two nuclear genes, MYH6 and FGB, a Bayesian statistical method, was used implemented in PHASE (Scheet & Stephens, 2006; Stephens et al., 2001) and in DnaSP v 5 (Librado & Rozas, 2009). The identified haplotypes were based on three independent runs for each gene using 1000 iterations.

### 3.4 Genetic variation

Genetic variation in MYH6, FGB, and complete mitochondria was estimated as haplotype diversity (HD) and nucleotide diversity (π) using DNAsp (Librado & Rozas, 2009) and ARLEQUIN version 3.5.1 (Excoffier & Lischer, 2010).

### 3.5 Detection of selection

Positive selection acting on the 13 coding mitochondrial genes and the two nuclear genes was tested using the MEME (Mixed Effects Model of Evolution; Murrell et al., 2012) and FUBAR (Fast Unbiased Bayesian AppRoximation; Murrell et al., 2013). These tests are codon‐based selection tests implemented in HyPHY (DataMonkey server, (https://www.datamonkey.org)). The methods test for episodic selection (MEME) or diversifying (positive) and negative selection (FUBAR) acting on individual codons. Default settings were used. Only codons shared among ≥2 individuals were used to ensure that potential sequencing errors did not affect the results, and their positions were visualized in the consensus phylogenetic tree estimated in *BEAST (see below).

### 3.6 Population demography, haplotype networks

Historical demographic population fluctuations were explored using the neutrality of mutations in the five different datasets, tested using Tajima's *D* test of selective neutrality (Tajima, 1989) and Fu's *F*_S_ (Fu, 1997). Excess numbers of low frequency mutations relative to expectations under the standard neutral model are indicative of recent population growth and are detected as significantly negative values of Tajima's *D* and Fu's *F*_S_. Statistically, significant positive values for *F_S_* indicate a deficit of rare haplotypes suggesting that the population has experienced a bottleneck.

The relationships among nuclear (MYH6 and FGB) and mitogenome haplotypes were estimated based on Median‐joining network to allow for intermediate haplotypes in the network for the mitogenomes (Bandelt et al., 1999). The network was generated using DnaSP (Librado and Rojas 2009) and POPART with ε set at zero (Leigh & Bryant, 2015).

### 3.7 Mutation rates estimation

Due to the ongoing debate concerning the use of mutation rates estimated from evolutionary old time points in the speciation history and the applicability of these rates to estimate divergence times between populations within a species (Ho et al., 2005; Subramanian & Lambert, 2011), two substitution‐rates were used to date observed splitting events. Consequently, divergence time was estimated based on a fast 7.3%/site/MYR (Lebedev et al., 2018), and a slow 2%/site/MYR (Cheng et al., 2019) CytB mutation rates for Dipodidae. Given these two mutation rates were estimated for CytB, we used the formula by Andersen et al. (2017) to estimate the mutation rates for the full mitogenome. The calculations were based on theta (θ) estimated in DNAsp 6.10.03 (Rozas et al., 2017), the assumption of no recombination and identical effective female population size (*N*_fe_ = θ/2μ) between the regions: μgene = ((θgene × μmitogenome)/θmitogenome)). The new estimated mutation rate for the full mitogenome based on the fast mutation rate was 5.813%/site/Myr, and 1.519%/site/Myr for the slow mutation rate.

### 3.8 Population structure

Population structure was analyzed using a pairwise Ф_ST_ test conducted on pairwise nucleotide differences in ARLEQUIN v3.5.2 (Excoffier & Lischer, 2010). The tests were run for 10,000 permutations over individual haplotypes among populations. Furthermore, we applied the non‐parametric method (Kolmogorov–Smirnov, (K–S) test) suggested by Carr et al. (2015) that analyze “the cumulative pairwise mismatch distribution between populations.” (Carr et al., 2015) The Kolmogorov–Smirnov test does not assume that data are sampled from a specific distribution, testing the null hypothesis that data originate from the same population with identical distribution. This was performed to analyze the phylogenetic and phylogeographic structure among the regions. The nucleotide variants from all mitogenomes within a geographical region (i.e., DK_North) were compared pairwise to the nucleotide substitution sites from another region (i.e., DK_South) based on the aligned mitogenomes. The cumulative frequency curve was formed for each and the largest, unspecified observed difference, D (the vertical offset of the curves), over the range of all differences between the cumulative frequency distributions was identified. The significance of the obtained maximum, D, of the pairwise comparison was assessed by Da, the critical value which has been shown (Kolmogorov, 1933) to be independent of the reference distribution. All the pairwise Kolmogorov–Smirnov tests were performed using the Web‐based statistical software, http://www.physics.csbsju.edu/stats/KS‐test.html. Due to very low sample size in three of the geographical areas (*N* < 5, Russia, Norway and Latvia), this analysis was only applied to the remaining six regions.

### 3.9 Phylogenetic analysis and divergence time estimation

Phylogenetic analysis was conducted on the mitogenome dataset to explore the evolutionary relationships and estimate Time of Most Recent Common Ancestor (TMRCA). First, to investigate the overall evolutionary relationships, a Neighbour joining (NJ) phylogeny was built in MEGA v 6.06 (Tamura et al., 2013) using the TrN substitution model and 1000 bootstraps. Then, Bayesian phylogenetic analysis and estimation of TMRCA was performed using BEAST v1.8.4 (Drummond & Rambaut, 2007). A concatenated dataset including all 13 mitochondrial coding genes was analyzed. The sequences were further divided into the three codon positions to allow separate estimation of mutation rate. The best substitution model was chosen based on a maximum likelihood search conducted in MEGA. Initial analysis found the HKY + G + I model to constitute the most accurate substitution model for the dataset. The final analysis was based on the simpler HKY model due to problems with MCMC convergence in BEAST when using the HKY + G + I model. However, the phylogeny and the estimated TMRCA of all major groups were extremely similar between analyses supporting a limited effect of the substitution models. Initial phylogenetic analysis showed the presence of three major clades. These may have different demographic histories, which could affect divergence estimates if not accounted for (Ho et al., 2008). Thus, to allow differences in population size changes, the final analysis was conducted in *BEAST (Heled & Drummond, 2010). This program uses two different tree priors: a speciation prior for between‐clade branch patterns and a coalescence prior for the within‐clade branch patterns, which allows inferences of different demographic history between lineages. Piecewise linear and constant root, piecewise linear and piecewise constant change models of effective population size were used as coalescence priors to compare the robustness of the divergence estimates given differences in demographic history. The YULE prior was used as the between‐clade prior. To estimate the best set of models, bayes factor calculation, as proposed by Suchard et al. (2001), was conducted in BEAST. All phylogenetic analyses used a constant clock as a preliminary test in BEAST showed no significant rate heterogeneity among branches. Both the slow and the fast mutation rates were used to estimate divergence time in years.

The final MCMC samples were based on a run for 50,000,000 generations, and genealogies were sampled every 5000 generations with 10% discarded as burn‐in. Examination of convergence and effective sample size (ESS) values was conducted using TRACER v1.5 (Drummond & Rambaut, 2007). All parameters had ESS values >200, and additional runs gave similar results. The maximum clade credibility tree with mean heights for branches was estimated in the program TREEANNOTATOR (Drummond & Rambaut, 2007) with 10% burn‐in and visualized and edited in the program FIGTREE v1.3.1 (Andrew Rambaut, University of Edinburgh, http://tree.bio.ed.ac.uk/software/figtree/).

Divergence time between the different geographical groups was estimated using IMa software (Hey & Nielsen, 2007) using the full mitogenomes. Only groups with more than five individuals were analyzed to avoid spurious results due to low sample size. IMa infer divergence time between two populations from a common ancestral population. It assumes constant population sizes and no intragenic recombination, which is not occurring in mtDNA. A model with isolation without migration (m=0) was chosen to mimic founding of the Danish populations. Population sizes (*q*) were set to 300 and divergence time, *t*, to 20. A geometric heating scheme with 15 MCMC chains with the parameters, g1 = 0.7, g2 = 0.9, L = 3.0 and a burn‐in = 30,000 was used to explore the parameter space. Convergence was evaluated by ESS and performing two runs for each pairwise estimation of divergence time showing similar results. The dataset was analyzed using the HKY substitution model and an inheritance scalar of 0.25 to account for the lower effective size of mtDNA compared with nuclear DNA. As in *BEAST, the fast and slow mutation rates were used for divergence time estimation in years.

## 4 RESULTS

### 4.1 Haplotype variation

Fifty‐four mitogenomes, 69 FGB and 66 MYH6 nuclear sequences of *S*. *betulina* and one *S*. *strandi* were used in the analysis (Table 1). In total, the 54 different mitogenome haplotypes were detected representing 1699 segregating sites. For the two nuclear genes, seven different sequences were observed in MYH6 with six segregating sites, and in FGB, ten sequences and eleven segregation sites were found after specifying the haplotypes in PHASE including *S*. *strandi* in both analyses (Scheet & Stephens, 2006; Stephens et al., 2001).

**TABLE 1:** Sample size (N), Hd (haplotype diversity), π (nucleotide diversity), SD (standard deviation), Tajima D and Fu's *F*_s_ (Fu, 1997) of the complete mitogenome dataset and for the two nuclear genes, FGB and MYH6, for all analyzed geographical regions of the Northern Birch Mouse

	DKNT	DKS	Sweden	Norway	Estonia	Latvia	Russia	Slovakia	S. Strandi
Mitogenomes									
N	16	10	8	4	5	1	4	5	1
H	16	10	8	4	5	1	4	5	1
Hd	1	1	1	1	1	NA	1	1	NA
SD	0.022	0.045	0.063	0.177	0.126	NA	0.177	0.126	NA
π %	2.218	0.614	2.212	1.457	3.728	NA	1.491	3.605	NA
SD %	0.223	0.144	0.466	0.371	0.783	NA	0.705	0.621	NA
Tajima D	1.476	−1.137	0.48	1.062	0.879	NA	−0.714	0.426	NA
Fu's FS	0.932	0.603	2.52	3.672	4.101	NA	3.694	4.065	NA
MYH6									
N	23	10	9	6	5	2	5	5	1
H	5	3	3	3	3	2	3	4	1
Hd	0.605	0.653	0.647	0.682	0.644	0.667	0.689	0.733	NA
SD	0.042	0.065	0.069	0.091	0.101	0.204	0.104	0.12	NA
π %	0.316	0.358	0.342	0.396	0.535	0.553	0.424	0.461	NA
SD %	0.038	0.049	0.053	0.064	0.084	0.169	0.068	0.092	NA
Tajima D	−0.38	1.18	1.02	1.29	0.78	1.89	1.44	0.17	NA
Fu's FS	−0.93	0.9	0.69	0.59	0.98	1.53	0.52	−0.66	NA
FGB									
N	24	10	9	6	5	2	5	7	1
H	3	3	4	2	4	3	3	3	1
Hd	0.301	0.353	0.549	0.303	0.644	0.833	0.6	0.538	NA
SD	0.112	0.123	0.126	0.147	0.152	0.222	0.131	0.115	NA
π %	0.053	0.074	0.133	0.051	0.185	0.282	0.124	0.099	NA
SD %	0.021	0.029	0.037	0.025	0.046	0.102	0.034	0.025	NA
Tajima D	−0.59	0.24	−0.28	−0.19	0.1	0.17	0.12	−0.2	NA
Fu's FS	−0.62	0.65	−0.67	−0.27	−0.69	−0.13	−0.1	−0.37	NA

Note

Bold = *p* < .05 (none). Analyses were conducted in DnaSP and ARLEQUIN (Excoffier & Lischer, 2010; Librado & Rozas, 2009).

DKNT, DK‐North; DKS, DK‐South.

The genetic diversity in terms of nucleotide diversity (π) among the mitogenome samples ranged from ~0.614% ± 0.144% observed in the DK_South (DKS) sample to ~3.728% ± 0.783% observed in the Estonia sample (Table 1). For the nuclear genes, nucleotide diversity for MYH6 ranged from ~0.316% ± 0.038% in DK_North (DKNT) to ~0.553% ± 0.169% in the Latvian sample. In the FGB gene, the nucleotide diversity ranged from ~0.053% ± 0.021% in DK_North and to ~0.282% ± 0.102% in the sample from Latvia. Sample size was, however, very low from Latvia.

### 4.2 Detection of selection

As expected, most non‐neutrally evolving codon positions were under strong purifying selection (d*N*/d*S* < 1; Supplementary S3). Evidence for significant episodic positive and diversifying selection estimated in MEME and FUBAR was identified in seven genes where the mutations in five genes caused a replacement in amino acids with extremely similar physiochemical properties (Supplementary S4). In three of the seven genes, MEME detected possible non‐conservative substitution (Supplementary S3, see also Figure 3). In ND2, one substitution was indicated (isoleucine (ATA) to glutamine (CAA) at site 933) which was observed in *S*. *strandi* and an individual from Slovakia (SLO3). These amino acids have different physiochemical properties indicated by rather high Grantham's distance which might influence protein function (Grantham, 1974). No significant selection was detected acting in the two nuclear genes (data not shown).

### 4.3 Population demography, haplotype relationship

Exploring the possibility of detecting signs of population expansion or bottlenecks, Tajima's D and Fu's *F_S_* were estimated for all datasets (Table 1). None of the two parameters were significant in any of the datasets used. This supported the assumption of constant population sizes, which is an essential assumption for the divergence time estimates conducted in IMa.

The median‐joining network (Supplementary S5) of the 54 genetically different mitogenomes displayed a huge genetic variation where the haplotypes were separated with one to 954 mutations. Two Danish individuals (DK_13S and DK_5NT) were separated with just one mutation. No clear strict pattern was observed. However, there was a tendency that mitogenomes from DK_South clustered together, which was also observed for mitogenomes from Sweden. Finally, mitogenomes EST_4, SLO_3, and SLO_8 all separated distinctly from the rest of the mitogenomes, clustering with *S*. *strandi*. Genetic species identification in BOLD (https://www.boldsystems.org/) using the ~700 bp mtDNA COI barcoding region confirmed the observed close genetic relationship to *S*. *strandi*. All three individuals showed the closest genetic relationship to *S*. *strandi* compared with other species of the genus (including *S*. *concolor*, *S*. *subtilis*, and *S*. *servertzovi*) with a sequence similarity of 98.92%, 96.76%, and 96.91% for SLO3, SLO8, and EST4. *S*. *betulina* was the species with the second highest sequence similarity (94.47%, 95.85%, and 96.14%), respectively. The *S*. *strandi* sequence analyzed as part of the study matched 100% with other *S*. *strandi* sequences and 93.66% to *S*. *betulina*.

The variation observed in the two nuclear genes (Supplementary S6a,b) was much lower and the haplotypes had to be inferred using PHASE. Hence, seven haplotypes were observed in MYH6. The two common sequences, SBdkMYH1 and SBdkMYH3, were shared amongst all locations, while SBdkMYH2 was not found amongst the Estonian and Latvian individuals. One sequence was observed only in Estonia and Slovakia (SLO_3 and EST_4, the same individuals that clustered close to *S*. *strandi* in the mitogenomes), while two unique sequences were observed in DK_North (Supplementary S6a).

In FGB, 10 haplotypes were detected. SBdkFGB1 was found in all locations while SBdkFGB2 was detected in all locations except Norway, Estonia, and Slovakia. SBdkFGB5 and SBdkFGB6 were found in Norway, Slovakia, and in Sweden, Slovakia, and Estonia, respectively. The remaining five were unique for DK_North, DK_South, Sweden, Russia, and Latvia. The sibling species *S*. *strandi* had unique sequences in all datasets (Supplementary S6b).

### 4.4 Population structure

Despite low sample sizes, the pairwise analysis of molecular variance (AMOVA) analysis performed on mitogenome‐variation between the different areas (including samples with *N* > 4) revealed significant Ф_ST_ estimates between southern Denmark and Estonia and Slovakia. For the two nuclear genes, only FGB revealed significant differences. Still, despite low sample sizes, Estonia differed from the two Danish locations while Slovakia was significantly different from DK_North (DKNT; Table 2). Testing only DK_North and DK_South not applying a sequential Bonferroni correction, a significant Ф_ST_ was observed (Ф_ST_ = 0.201, *p* = .011). This was not observed when testing the two nuclear genes using an identical approach (Ф_ST‐FBG_ 0 0.002, *p* = .326; Ф_ST‐MYH6_= −0.021, *p* = .665).

**TABLE 2:** Pairwise population structure estimates, Φ_ST_, (below diagonal) based on pairwise differences of complete mitogenomes and Φ_ST‐FGB_ (above diagonal) based on sequence variation of the nuclear FGB gene for the different locations (ARLEQUIN 3.5.1; Excoffier & Lischer, 2010)

	DKNT	DKS	Sweden	Estonia	Slovakia
DKNT	0	0.002	0.097	**0.463**	**0.267**
DKS	0.201	0	0.061	**0.32**	0.128
Sweden	−0.017	0.137	0	0.255	0.148
Estonia	0.009	**0.399**	0.035	0	0.1
Slovakia	0.045	**0.358**	0.046	−0.139	0

Note

Bold = significant after sequential Bonferroni correction, α = 5% (Rice, 1989).

**FIGURE 2:**
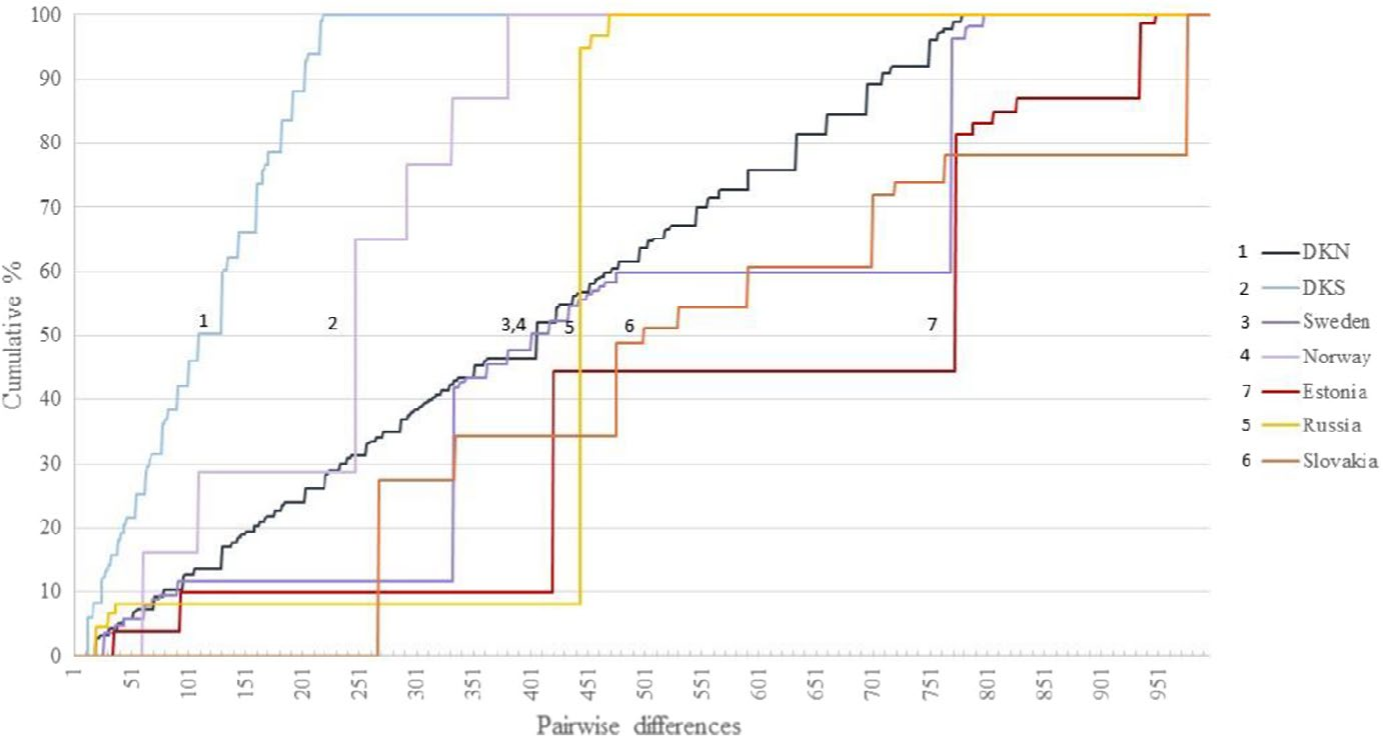
Cumulative pairwise distribution curves for the Kolmogorov‐Smirnov test based on the nucleotide differences between the mitogenomes sampled in the seven locations of Northern Birch Mouse. Norway and Russia are included for illustration despite the low sample sizes

The results of the pairwise Komolgorov–Smirnov tests showed a significant offset of the cumulative distribution curves (Table 3; Figure 2) for DK_South (DKS) and all the other four regions, including DK_North (DKNT). Furthermore, the offset of the DK_North curve was significantly different from Estonia and Slovakia, and finally, the offset of the Swedish curve was significantly different from the Slovakian curve (Table 3; Figure 2).

**TABLE 3:** Results of the pairwise non‐parametric Kolmogorov–Smirnov tests between the cumulative pairwise distribution of nucleotide differences within *Sicista betulina* mitogenomes sampled in Denmark, Sweden, Estonia, and Slovakia

	DKNT	DKS	Sweden	Estonia
DKS	**0.716**			
Sweden	0.256	**0.714**		
Estonia	**0.582**	**0.8**	0.493	
Slovakia	**0.533**	**1**	**0.621**	0.5

Note

Bold = significant after sequential Bonferroni correction, α = 5% (Rice, 1989).

### 4.5 Phylogeny and divergence time estimation

**FIGURE 3:**
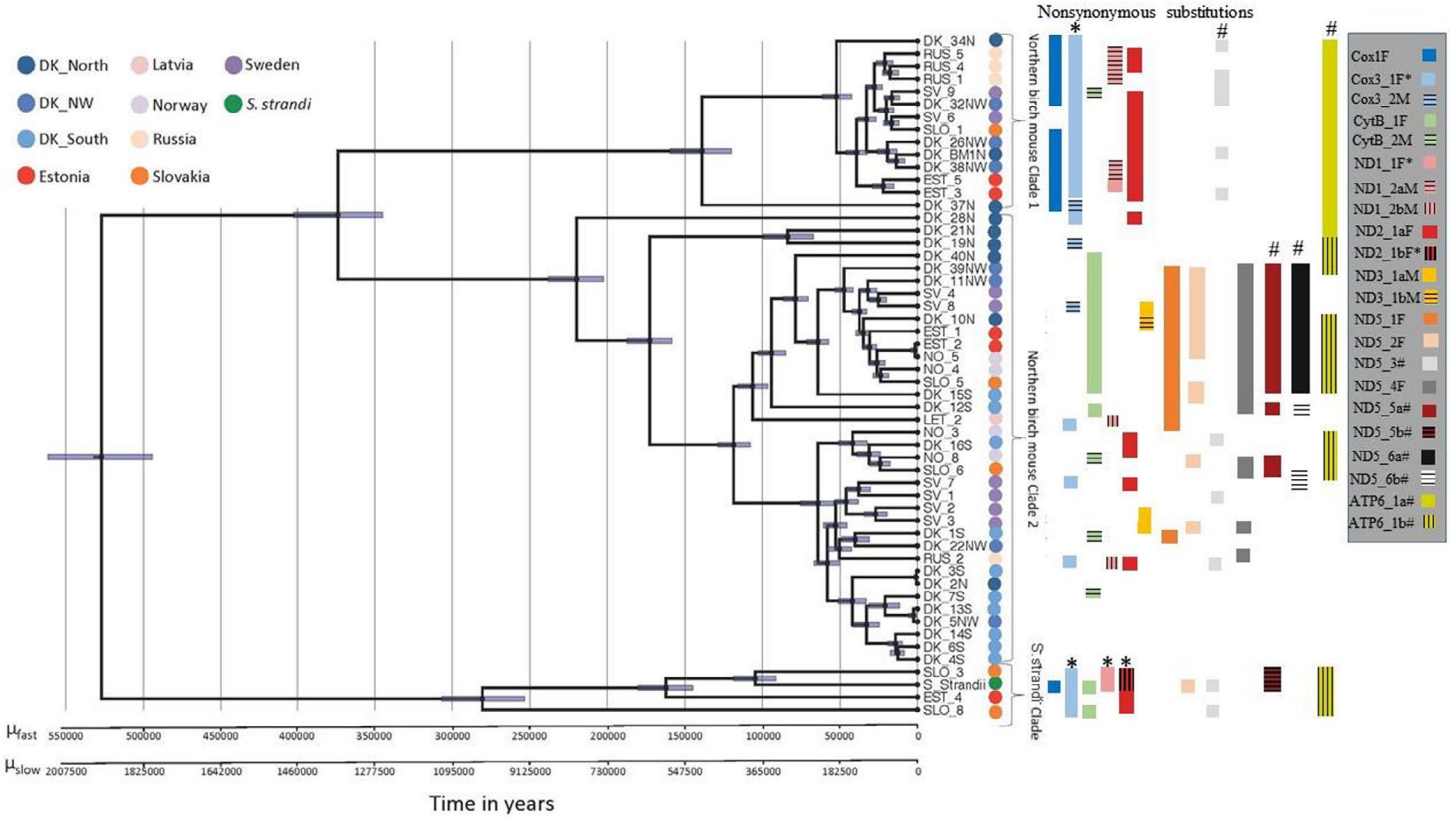
Phylogenetic tree based on the coding regions of mtDNA of all Northern Birch Mouse samples and one *Sicista strandi*. Bars represent the 95% lower and upper bound of the posterior density (HPD) interval. Mutation rates are assumed constant and unscaled estimates of divergence time (Time of Most Recent Common Ancestor, TMRCA) are obtained using two different rate estimates to account for possible time‐dependency. The slow and fast mutation rates (μSLOW, μFAST) are both derived from studies on Dipodidae (Cheng et al., 2019; Lebedev et al., 2018) and calculated for the full mitogenome in the present study (slow rate = 1.59%/site/MYR, fast rate = 5.81%/site/MYR). For nonsynonymous changes detected in FUBAR and MEME numbers designate sites of the mutation. Letters after numbers indicates another mutation at that site as indicated with the shading. Vertical and horizontal shading designate different mutations. *Positive sites obtained from selection test performed in MEME at another site compared to the test conducted in FUBAR and observed in exactly the same individuals. ^#^Positive sites and mutations observed in exactly the same individuals as obtained with the selection test conducted in FUBAR. In the legend showing the genes with positive sites for selection, F = FUBAR and M = MEME

The three different Bayesian analyses showed very similar estimates of TMRCA. However, the analysis using the piecewise linear and constant root coalescence prior showed the best fit to the data (bayes factor >9000) and were therefore used for visualization of evolutionary relationships and reporting of TMRCA (Figure 3). One clade corresponded to *S*. *strandi*, which showed a close evolutionary relationship with three individuals of Northern Birch Mice collected from Slovakia (SLO_3 and SLO_8) and Estonia (EST_4) thus supporting the results of the median‐joining network tree for those individuals. The two last clades only included Northern Birch Mouse individuals. Clade 1 constituted a smaller clade of individuals from Russia, Slovakia, Estonia, Sweden, and DK_North. The second clade included individuals from Russia, Slovakia, Estonia, Sweden, Norway, DK_North, and all the individuals from DK_South. Whether the distribution of individuals between the two last clades was significantly different between DK_South and DK_North was tested using the Monte Carlo Chi‐square method in REAP 4.0 (McElroy et al., 1991). This procedure tests the haplotype heterogeneity and haplotype distribution amongst the areas based on 1000 simulations. The test showed a marginal significant heterogeneity amongst the areas (*p* = .048) suggesting that the distribution amongst the clades might not be attributed to the small sampling size.

TMRCA between the clades including the sister‐species *S*. *strandi* and the two clades of Northern Birch Mouse was estimated to 527,033 years (494,207–561,346 95%HPD) when using the fast substitution rate and ~three times higher when using the slow substitution rate. Divergence time between the two clades of Northern Birch Mouse was estimated to 373,837 years (345,064–402,173 95%HPD) and 1,364,505 years (1,259,484–1,467,931 95%HPD; Figure 3). TMRCA for the three clades was estimated to 280,651 (253,437–306,927 95%HPD) for the *S*. *strandi* clade, 138,998 years (119,902–159,835 95%HPD) for clade 1, and 219,873 years (202,598–238,324 95%HPD) for clade 2 of Northern Birch Mouse using the fast substitution rate. Using the slow substitution rates, the estimates were 1,024,376 years (925,045–1,120,284 95%HPD) for the *S*. *strandi* clade and 507,343 years (437,642–583,398 95%HPD) and 802,537 years (739,483–869,883 95%HPD) for clade 1 and 2, respectively. The estimated substitution rate was higher for the 3^rd^ codon position, followed by the 1st and 2nd positions (relative values from BEAST for 1, 2 and 3 base positions: 0.584, 0.242, and 2.174).

**FIGURE 4:**
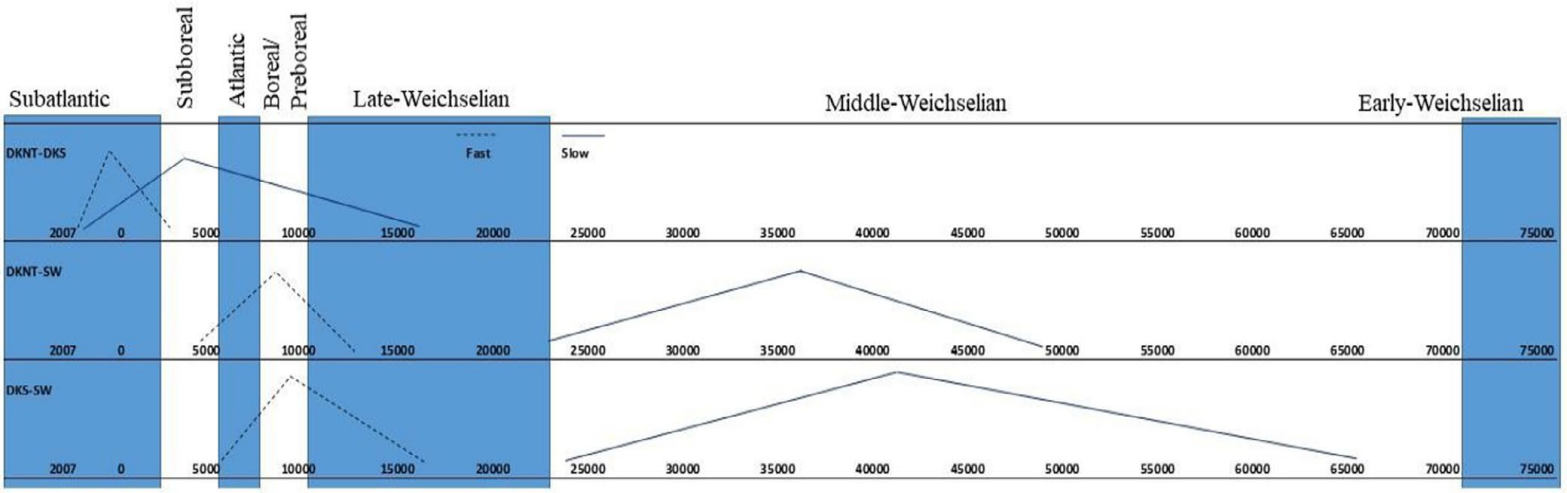
The probability of divergence time estimated between the localities with the highest sample sizes, Denmark and Sweden performed in IMa (Hey & Nielsen, 2007). Stippled lines—represents estimates using the fast mutation rate (5.81%/site/MYR, present study), and solid lines represents estimates using the slow mutation rate, 1.58%/site/MYR (present study). Results shown for samples with >5 individuals

Focusing on the Danish populations, the population divergence time estimates using IMa (Hey & Nielsen, 2007) suggested a split between DK_North and DK_South occurring around ~534 AD (3053 BC–1997 AD) for the fast mutation rate while the slow mutation rates gave a spilt around six times higher (Table 4; Figure 4). Only samples from Sweden were further included in the analysis due to the small sample sizes from other countries. The estimated divergence time between the two Danish samples and Sweden was at the same level occurring around 8656 BC (12,103 BC–4959 BC) and 9889 BC (16,458 BC–5314 BC) using the fast mutation rate (Table 4; Figure 4). The different estimates should, however, be interpreted cautiously given the uncertainty regarding low sample sizes and mutation rates. This is furthermore reflected in the width of the peaks in the probability of divergence (Figure 4) and the 90% high point density interval (HPD, Table 4).

**TABLE 4:** IMA results estimated without migration (Hey & Nielsen, 2007) shown for the samples with more than five individuals

Pop. 1	Pop. 2	Divergence time in years	
		µ: 7.3%/site/MYR^a^	µ: 2%/site/MYR^b^
DKNT	DKS	534 AD (3053 BC–1997 AD)	3368 BC (16,463 BC–1969 AD)
DKNT	Sweden	8656 BC (12,103 BC–4959 BC)	36,915 BC (49,595 BC–23,420 BC)
DKS	Sweden	9889 BC (16,458 BC–5314 BC)	41,413 BC (65,392 BC–24,716 BC)

Note

The values in brackets denote 90% High Point Density (HPD) intervals for the estimation.

^a^ Lebedev et al. (2018).

^b^ Cheng et al. (2019).

## 5 DISCUSSION

This study is the first to analyze the phylogeographic history of the Northern Birch Mouse in Northern Europe based on the complete mitochondrial genome. Overall, we find extremely high mitogenome diversity with all sequences being unique. This finds support in other studies of the family Dipodidae and may reflect a combination of short female migratory distances and a highly fragmented habitat. We show the presence of two distinct evolutionary linages in Northern Europe, including Denmark. The two species, *S*. *betulina* and *S*. *strandi*, diverged within the mid‐Pleistocene period identified as the Chibanian period (The Geological Society of Japan, “The Quaternary part of the International Chronostratigraphic Chart”). Within *S*. *betulina*, two evolutionary lineages diverged within the Elster glaciation period in mid‐Pleistocene. The two linages were observed in all analyzed areas, except southern Denmark (DK_South) which only represented clade 2. This suggests an allopatric origin and vicariance followed by secondary contact throughout its northern range. While the precise timing of this secondary contact cannot be inferred from this study, it likely occurred at an early stage either following or before the LGM. We find no evidence for repeated colonization's explaining the observed fragmented distribution of the species today. The origin of the southern Danish population is puzzling. The low mitogenome diversity and recent divergence from northern Denmark indicate that it may have been founded from northern Denmark. The results might also be ascribed to a combination of climatic changes and anthropogenic activities that have prompt modifications in landscape vegetation in southern Denmark reducing population size and increasing genetic drift or alternatively, possible mitogenome sampling bias. Finally, no strong evidence for positive selection acting on either mitochondrial or nuclear genes, which in general were characterized by the presence of strong purifying selection, were found. The few sites within the mitogenome with d*N*/d*S* > 1 was limited to amino acid changes with similar physiochemical properties suggesting neutral or relaxed purifying selection. This finding, combined with the observation of similar phylogeographic patterns between the mitogenome and nuclear markers, supports near neutrality of the mitogenome and excludes a significant influence of positive selection on the observed phylogeographical patterns, which could otherwise impede reliable phylogeographic assessment and estimation of divergence time (Ballard & Whitlock, 2004; Galtier et al., 2009).

### 5.1 Genetic variation

Genetic diversity was higher for the mitogenomes compared with the nuclear markers. This is expected as mitochondrial DNA in general evolves at a faster rate than nuclear genes and hence accumulates more mutations overtime and during rapid radiation (e.g., Matocq et al., 2007; Yue et al., 2015). Nevertheless, it was surprising to observe that every individual carried a unique mitogenome. This high genetic variation is also observed in other Dipodidae. Ben Faleh et al. (2012) observed 35 different CytB ‐haplotypes sequencing 45 *Jaculus orientalis* specimens, and Boratynski et al. (2012) detected 18 and 21 different CytB‐haplotypes among 21 samples of *Jaculuc deserti* and *Jaculus jaculus*, respectively. Furthermore, Fedyk et al. (2011) detected variation at the chromosomal level (2*n* = 32) where chromosome pair 2 was found to be polymorphic, composed of acrocentric (a) or biarmed chromosomes. Two variants of the biarmed chromosome were observed, a submetacentric (sm) and a subtelocentric (st). This polymorphism was detected in 17 samples of Northern Birch Mouse collected in lowland (6 st/st, 3 a/a, 1 a/st, 1 st/sm, 1 sm/sm) and mountain populations (2 sm/sm, 1 st/st, 1 st/sm, 1 a/sm) in Poland. Hence, suggesting that the st variant were predominant in the lowland and sm in the mountains (Fedyk et al., 2011). Thus, it seems as the family Dipodidae is highly polymorphic, which might reflect the rapid radiation of Rodentia during Paleocene/Early Eocene or might be influenced by introgression from other closely related species (Marivaux et al., 2004; Pisano et al., 2015; Yue et al., 2015).

The central‐marginal hypothesis predicts a decrease in genetic diversity with increasing distance from the glacial refugium (Eckert et al., 2008). This implies that the Danish and Swedish locations are expected to hold a lower nucleotide diversity compared with more southern and eastern locations such as Slovenia and Estonia. Despite the low sample sizes, this tendency was tentatively suggested by the observed variation in both the mitogenomes and the nuclear genes. As such, this study suggests recolonization of northern Europe from more southern (Carpathian) or eastern areas that served as refugia during the latest ice age. This is supported by many phylogeographical studies of steppe fauna which find higher genetic diversity in Southern and Eastern Europe (Kajtoch et al., 2016; Marková et al., 2020).

### 5.2 Selection and mutation rate

The 13 mitochondrial coding genes were observed mainly to be under purifying selection as observed in other studies (Andersen et al., 2017; Jacobsen et al., 2015; Tomasco & Lessa, 2011). However, some sites showed significant d*N*/d*S*‐values >1, suggesting possible positive selection. These non‐synonymous sites were mainly found in the ND genes, which in general show higher d*N*/d*S* than other mitochondrial genes (Andersen et al., 2017), and almost exclusively included situations where amino acid substitutions occurred independently in multiple places in the phylogenetic tree. ND genes belong to the electron transport system I (ETS I) in the four components of the mitochondrial ETS and have been observed having a faster mutation rate compared with the other three ETS (Matosiuk et al., 2014; Zhang & Broughton, 2013). This may be the result of functional constraints, as ND genes probably are of less importance for metabolic output than the other mitochondrial genes, which may lead to relaxed purifying selection (Blier et al., 2006). Positive selection was not further supported as the amino acid replacements showed very similar physiochemical properties, which suggests either neutral or near neutral changes. The same pattern was also observed in the four other genes with detected candidate codon positions for positive selection (COX1, COX3, CytB, and ATP6). Hence, while these could cover positive selected sites, we find it more parsimonious that the observations are a result of relaxed purifying selection and reflect neutrally or slightly deleterious mutations. Evidence for this has been found in many other mitogenome studies and may relate to change in the efficiency of purifying selection (Ho et al., 2005; Jacobsen et al., 2015), which might need time to remove slightly deleterious mutations. As a result, the observed mutation rate may also be higher, which supports the use of the fast mutation rate for divergence estimation. The rate was estimated to 5.81% substitutions/site/Myr based on a CytB mutation rate of 7.3%/site/Myr in rates for Dipodidae (Lebedev et al., 2018). This rate is slightly faster than the mitogenome mutation rate of 3.2%/site/Myr observed in *M*. *musculus domesticus* from the Kerguelen Archipelago (Hardouin & Tautz, 2013).

### 5.3 Population structure and demographic history

The prevalent phylogenetic methods used in population genetic studies have problems when all individuals are genetically different as in this study. The pairwise AMOVA based on differences between haplotypes/mitogenomes (Ф_ST_) among the populations is too conservative as all the variance is observed among the individuals. Consequently, the population structure pattern is difficult to resolve and only DKS was found significantly different from the more distant localities (Estonia, Slovakia, Russia). Using nuclear markers with slower mutation rates on the other hand revealed too little variation. Only FBG was able to detect significant genetic differentiation between the more distant locations, Slovakia and Estonia, and the Danish locations (except Slovakia‐DKS). Due to the very high mitogenome variation, we chose to use the non‐parametric K–S test to analyze the population relationship and phylogenetic history as suggested by Carr et al. (2015). These authors used the non‐parametric K–S test to analyze phylogenetic history/structure of harp seals, *Pagophilus groenlandicus*, due to the observation that all the collected harp seals (*N* = 53) represented a unique mitogenome. The test compares the maximum difference (D) in time elapsed for the different populations to attain a certain level of phylogenetic diversity using the cumulative pairwise differences (Figure 2). Focusing at the 50% cumulative difference at the *y*‐axis, the significant offsets indicated that Estonia and Slovakia were different from the Danish and Swedish populations. Furthermore, the ranking (although sample sizes were too small to test for some of the populations) suggested that DK_South (DKS) < DK_North (DKNT) – Sweden < Slovakia < Estonia where DK_North and Sweden indicated comparable phylogenetic histories and ages. Slovakia and Estonia contained the mitogenomes having accumulated the highest mutation numbers implying despite rather low sample sizes that they represented the oldest populations analyzed. Interestingly, the southern Danish population (DK_South) compared with northern Danish population (DK_North) indicated a different phylogenetic history, ranking the phylogeographic structure and age of the populations. The fewer pairwise nucleotide differences accumulating in shorter time indicated that the southern Danish population (DK_South) might be recently founded following a bottleneck/founding event, parallel to a star‐like haplotype pattern observed in a haplotype network (Slatkin & Hudson, 1991). The differences between DK_South and DK_North reflect the phylogenetic signal where DK_South does not include sequences within clade 1. While this is expected if the population is recently founded, it may also represent possible climatic changes or anthropogenic activities leading to modifications in landscape vegetation in the southern part of Denmark (DK_South), reducing population size and increasing genetic drift. Nonetheless, the pattern is different for the two nuclear genes where DK_South show higher but comparable genetic diversity compared with DK_North. It is possible that this can be ascribed to the lower effective size of the mitogenome making it more accessible to genetic drift than the nuclear genes. On the other hand, it cannot be excluded that the mitogenome result reflects the small sampling size and hence potential sampling bias, as indicated from by the MCMC based Chi‐square test in REAP (McElroy et al., 1991) that only showed marginal significance.

### 5.4 Phylogeograpic patterns and divergence times

In general, the genetic relationship reflected by the median‐joining haplotype network based on mitogenomes supported a closer relationship between individuals within the different locations compared to between locations. However, the huge number of mutations separating the individuals even within the same geographical area support the hypothesis that colonization of Northern Europe might have occurred from different refugia. This combined with limited mobility of the species, repeated changes in climate and vegetation together with rapid radiation connected to Rodentia are probably the drivers behind the observed genetic pattern.

The nuclear haplotype network reflected a very close relationship between all locations illustrating the limited observed variation in those genes prompt by an expected lower nuclear mutation rate and shorter sequences leading to a lower genetic resolution. Nevertheless, both genes showed patterns in support of expansion from several refugia.

The phylogenetic tree estimated TMRCA between the *S*. *strandi* clade and the Northern Birch Mouse clades to ~527,033 years ago (494,207–561,346 95%HPD) using the fast substitution rate (µ_fast_). This is slightly more recent compared with the divergence time, ~670,000 years, suggested by Rusin et al. (2018) based on variation in CytB and five nuclear genes but earlier than the around 1 Mya (Early Pleistocene) estimated by Lebedev, Rusin, et al. (2019) and well within the suggested appearance of *S*. *betulina* from the fossil record in southeastern France ~781,000 to 126,000 years ago (Rofes et al., 2012). The differences in the TMRCA estimates can probably be attributed to the different kind and number of markers (nuclear and/or mitochondrial genes), and mutation rates used in the different studies. Common for these studies are that they support the species status of *S*. *betulina* and *S*. *strandi*. Interestingly, we found that three individuals from Slovakia and Estonia actually cluster with the *S*. *strandi* in the mitogenome phylogeny. Assuming that identification of the specimens is correct, this suggests possible introgression between *S*. *strandi* and *S*. *betulina* occurring as recent as ~100,000 years ago. The finding of the nuclear analysis further supports this possibility, as the observed haplotypes in the three individuals does not match the nuclear haplotype found in *S*. *strandi*, but instead matched sequences similar to other Northern Birch Mouse specimens. This finding seemingly excludes morphological species misidentification of these individuals, but can be explained by potential gene flow. Hence, more samples are needed to investigate the extend and direction of possible introgression events in future studies including other *Sicista* species in the area, that is *S*. *subtilis* and *S*. *trizona*.

Focusing on the divergence time estimates obtained for the Fennoscandinavian populations (*N* > 5), Danish and Swedish populations might have diverged around Preboreal/Boreal (11,653–8000 cal BP). This period was characterized by rapid climate changes (Jessen et al., 2015). Temperature and precipitation fluctuated rapidly impacting the vegetation and fauna. The vegetation developed from tundra into open boreal birch‐pine‐dominated forests and further into forests consisting of more warmth demanding deciduous trees. Consequently, tundra‐forest‐adapted species such as the Northern Birch Mouse might at a distance have followed the northwards contracting ice edge, probably crossing the land bridge connecting eastern Denmark to Sweden (Aaris‐Sørensen, 2016; Jessen et al., 2015; Wygal & Heidenreich, 2014). Also, vegetated riverbanks might have acted as dispersal routes north as suggested by Pilats and Pilate (2009). A wide strait between Denmark and Sweden was formed shortly after, leading to isolation between Danish and Swedish populations (Bennike et al., 2012).

Divergence estimation between DK_North and DK_South suggested divergence during Early Neolithic to Late Iron Age (400 AD–1050 AD) depending on mutation rate (Odgaard & Rasmussen, 2000). In general, the landscape was influenced by anthropogenic activities and climatic changes during these time periods causing changes in forest land cover, the type of forest and the surrounding land use, increasing the grassland and heathland cover which probably have determined the distribution of the Northern Birch Mouse (Nielsen & Odgaard, 2010). However, the result remains tentative as the small sample size prevents accurate analysis of population structure and divergence timing. Thus, whether the two Danish areas indeed represent two recently reproductively isolated populations cannot be fully demonstrated by this study and we therefore recommend additional genetic studies using larger sample sizes and potentially more genetic markers to investigate this possibility.

## CONFLICT OF INTEREST

The authors declare no competing interests.

## AUTHOR CONTRIBUTION

**Liselotte Wesley Andersen:**Conceptualization (equal); Data curation (equal); Formal analysis (equal); Investigation (equal); Methodology (equal); Project administration (equal); Writing – original draft (equal); Writing – review & editing (equal). **Magnus W Jacobsen:** Conceptualization (equal); Formal analysis (equal); Investigation (equal); Methodology (equal); Project administration (equal); Writing – original draft (equal); Writing – review & editing (equal). **Jane Frydenberg:** Formal analysis (equal); Methodology (equal). **Julie Dahl Møller:** Data curation (equal); Methodology (equal); Writing – review & editing (equal). **Thomas Secher Jensen:** Conceptualization (equal); Funding acquisition (equal); Writing – original draft (equal); Writing – review & editing (equal).

## Supporting information

Supinfo S1

## Data Availability

All sequences are uploaded and archived in GenBank with the accession numbers: MZ555638–MZ555647(FBG), MZ555631–MZ555637(MYH6), MZ562682, MZ570912–MZ570964(mitogenomes).
