# CORRIGENDUM

**DOI:** 10.1002/ece3.8968

**Published:** 2022-05-24

In the recent article by Andersen et al. (2022), the word "MYH6" should be replaced by "MYH7" throughout the paper.

The authors apologize for the error.

## References

[ece38968-bib-0001] Andersen, L. W. , Jacobsen, M. W. , Frydenberg, J. , Møller, J. D. , & Jensen, T. S. (2022). Phylogeography using mitogenomes: A rare Dipodidae, *Sicista betulina*, in North‐western Europe. Ecology and Evolution, 12, e8865. 10.1002/ece3.8865 35475180 PMC9022092

