# Supplementary material for: Phylogeography using mitogenomes: A rare Dipodidae, Sicista betulina, in North‐western Europe
Source: Ecol Evol. 2022 Apr 21;12(4):e8865. doi: 10.1002/ece3.8865 (PMC9022092; doi:10.1002/ece3.8865)
Supplement: Supplementary file 1 — Supinfo S1 [file ECE3-12-e8865-s001.docx]

Supplementary 1. Distribution map of the three Birch Mouse species, *Sicista betulina*, *Sicista subtilis* and *Sicista standi* according to the information given in the IUCN database.


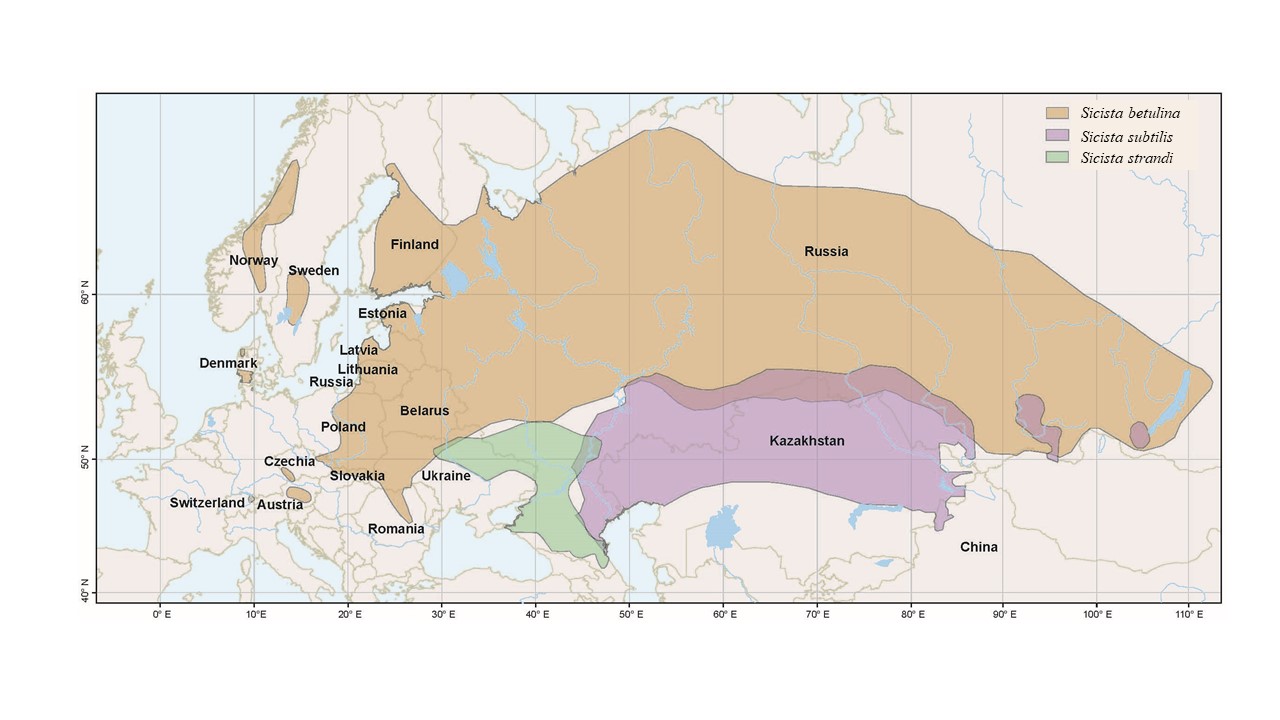


Supplementary 2. Primers used to amplify the two nuclear genes and complete mitogenome in the Northern Birch Mouse and *Sicista strandi* and the fragment length (size). The primers for the mitogenome were developed from *Microtus rossiaemeridionalis* (DQ015676.1) and *Apodemus agrarius* (HM034866.1) (Triant and Dewoody 2006)

|  | | | | | |
| --- | --- | --- | --- | --- | --- |
| Locus |  |  |  |  | Size |
| MYH6 | MYH2f | GAA CAC CAG CCT CAT CAA CC | | | 241bp |
|  | MYH2r | TGG TGT CCT GCT CCT TCT TC | | |  |
| FGB | β17-mammL | ACC CCA GTA GTA TCT GCC GTT TGG ATT | | | 591bp |
|  | βfib-mammU | CAC AAC GGC ATG TTC TTC AGC AC- | | |  |
| mtDNA 1 | P7f | CCC CTT ATG AAT GCG GCT TTG AC | | | ~3.6kb |
|  | P8r | CAG TAA GGG CTA GGC TGC CA | | |  |
| mtDNA 2 | P3f | CAG AAG TTC AAA TCC TCT CCT TA | | | ~7.1kb |
|  | P6r | TGT ACT ATT CTT CTC ATT CTA | | |  |
| mtDNA 3 | P9f | ACC CAC GCA TTC TTY AAR GC | | | ~7.1kb |
|  | P2r | AAT GGT TGT AAA ATT CCG TAT GGT CCT AC | | | |

Supplementary 3. Results of tests of selection conducted in MEME (Murrell et al. 2012) and FUBAR (Murrell et al., 2013) for the 13 genes. Analyses were conducted in HYPhy (https://www.datamonkey.org) using all mitogenomes generated in this study using default parameters. Only mutations found in ≥2 individuals are shown.

| Gene | Number of sequences | Number of different haplotypes | Best substitution model | MEME |  | FUBAR | | |  |
| --- | --- | --- | --- | --- | --- | --- | --- | --- | --- |
|  |  |  |  | Positive sites | p-value | Positive sites | Probability ranges positive sites | Negative sites | Probability ranges negative sites |
| COX1 | 54 | 34 | GTR | 0 | - | 1 | >0.95 | 84 | >0.95 |
| COX2 | 54 | 17 | GTR | 0 | - | 0 | - | 20 | >0.95 |
| COX3 | 54 | 33 | GTR | 1 | <0.05 | 1 | >0.95 | 23 | >0.95 |
| CYTB | 54 | 28 | GTR | 1 | < 0.05 | 1 | >0.95 | 67 | >0.95 |
| ND1 | 54 | 44 | GTR | 2 | ≤ 0.05 | 1 | >0.95 | 73 | >0.95 |
| ND2 | 54 | 31 | GTR | 1 | <0.05 | 1 | >0.95 | 42 | >0.95 |
| ND3 | 54 | 21 | GTR | 1 | <0.05 | 0 | - | 10 | >0.95 |
| ND4 | 54 | 32 | GTR | 0 | - | 0 | - | 3 | >0.95 |
| ND4L | 54 | 9 | GTR | 0 | - | 0 | - | 0 | - |
| ND5 | 54 | 51 | GTR | 3 | <0.05 | 6 | >0.95 | 40 | ≥0.95 |
| ND6 | 54 | 28 | GTR | 0 | - | 0 | - | 15 | >0.95 |
| ATP6 | 54 | 25 | GTR | 1 | ≤ 0.05 | 1 | >0.95 | 25 | ≥0.95 |
| ATP8 | 54 | 11 | GTR | 0 | - | 0 | - | 3 | >0.95 |

|  |  |
| --- | --- |
|  | |

Supplementary 4. The amino acid replacements in the seven genes identified in the selection test conducted in MEME and FUBAR (HYPhy, https://www.datamonkey.org).

| MEME |  |  |  |  |  |  | FUBAR |  |  |  |  |
| --- | --- | --- | --- | --- | --- | --- | --- | --- | --- | --- | --- |
| Gene | Codon position | | No. of ind. | Codon | Amino acid | Gene | Codon position | | No. of ind. | Codon | Amino acid |
| Cox3 | 250 | 252 | 19 | ATC | ile | Cox1 | 169 | 171 | 14 | GTT | val |
|  |  |  | 34 | GTA | val |  |  |  | 40 | ATT | ile |
|  |  |  | 1 | ATA | ile | Cox3 | 523 | 525 | 21 | GCC | ala |
| CYTB | 952 | 954 | 50 | CGC | arg |  |  |  | 33 | ACC | thr |
|  |  |  | 4 | GGC | gly | CYTB | 958 | 960 | 14 | ATC | ile |
| ND1 | 7 | 9 | 45 | CTC | leu |  |  |  | 40 | CTC | leu |
|  |  |  | 5 | CCC | pro | ND1 | 280 | 282 | 51 | GCC | ala |
|  |  |  | 4 | TTC | phe |  |  |  | 3 | AGC | ser |
|  | 280 | 282 | 51 | GCC | ala | ND2 | 967 | 969 | 34 | TTA | leu |
|  |  |  | 3 | AGC | ser |  |  |  | 18 | ATA | ile |
| ND2 | 931 | 933 | 52 | ATA | ile | ND5 | 1618 | 1620 | 13 | CTT | leu |
|  |  |  | 2 | CAA | gln |  |  |  | 41 | TTT | phe |
| ND3 | 244 | 246 | 50 | AGC | ser |  | 1627 | 1629 | 13 | TCA | leu |
|  |  |  | 3 | AAC | asn |  |  |  | 41 | ACA | thr |
|  |  |  | 1 | CTC | leu |  | 1729 | 1731 | 12 | GCA | ala |
| ND5 | 31 | 33 | 53 | ATT | asn |  |  |  | 42 | ACA | thr |
|  |  |  | 1 | ATA | ile |  |  | 1761 | 38 | ATC | ile |
|  | 1183 | 1185 | 53 | ATG | met |  |  |  | 16 | ATA | ile |
|  |  |  | 1 | ACC | arg |  | 1783 | 1785 | 2 | GTA | val |
|  | 1729 | 1731 | 12 | GCA | ala |  |  |  | 39 | GCA | ala |
|  |  |  | 42 | ACA | thr |  |  |  | 12 | ATA | ile |
|  | 1783 | 1785 | 2 | GTA | val |  |  |  | 1 | ACA | thr |
|  |  |  | 39 | GCA | ala |  | 1795 | 1797 | 41 | ATC | ile |
|  |  |  | 12 | ATA | ile |  |  |  | 3 | ACC | thr |
|  |  |  | 1 | ACA | thr |  |  |  | 10 | GCC | ala |
|  | 1795 | 1797 | 42 | ATC | ile | ATP6 | 145 | 147 | 13 | ATA | ile |
|  |  |  | 3 | ACC | thr |  |  |  | 21 | GTA | val |
|  |  |  | 9 | GCC | ala |  |  |  | 20 | TTA | leu |
| ATP6 | 145 | 147 | 13 | ATA | ile |  |  |  |  |  |  |
|  |  |  | 21 | GTA | val |  |  |  |  |  |  |
|  |  |  | 20 | TTA | leu |  |  |  |  |  |  |
|  | 583 | 585 | 53 | ATT | ile |  |  |  |  |  |  |
|  |  |  | 1 | TTT | phe |  |  |  |  |  |  |

Supplementary 5. Median‐joining haplotype network of the mitogenomes between Northern Birch Mouse from Denmark, Sweden, Norway, Estonia, Latvia, Russia and Slovakia together with *S. strandi* indicating the haplotype relationships estimated using DnaSP (Librado and Rozas 2009) and POPART (Leigh and Bryant 2015). The numbers indicated on the line connecting haplotypes show the number of mutations separating the haplotypes (only mutations >20 is shown).


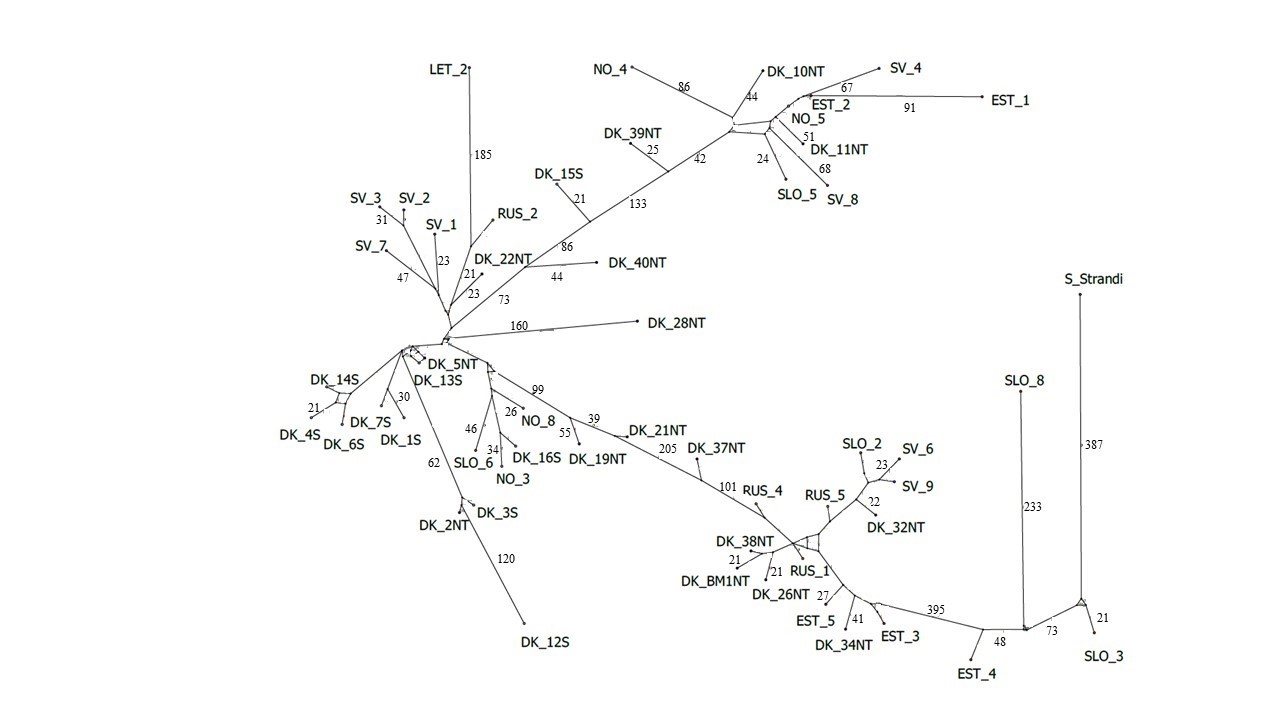


Supplementary 6. Median‐joining haplotype network of a) beta-myosin heavy-chain (MYH6) haplotypes and b) β-fibrinogen (FGB) haplotypes between Northern Birch Mouse from Denmark, Sweden, Norway, Estonia, Latvia, Russia and Slovakia together with *S. strandi* indicating the phylogenetic relationships estimated using DnaSP (Librado and Rozas 2009) and POPART (Leigh and Bryant 2015). The number of crossbars on the line connecting haplotypes indicates the number of mutations separating the haplotypes.

a)


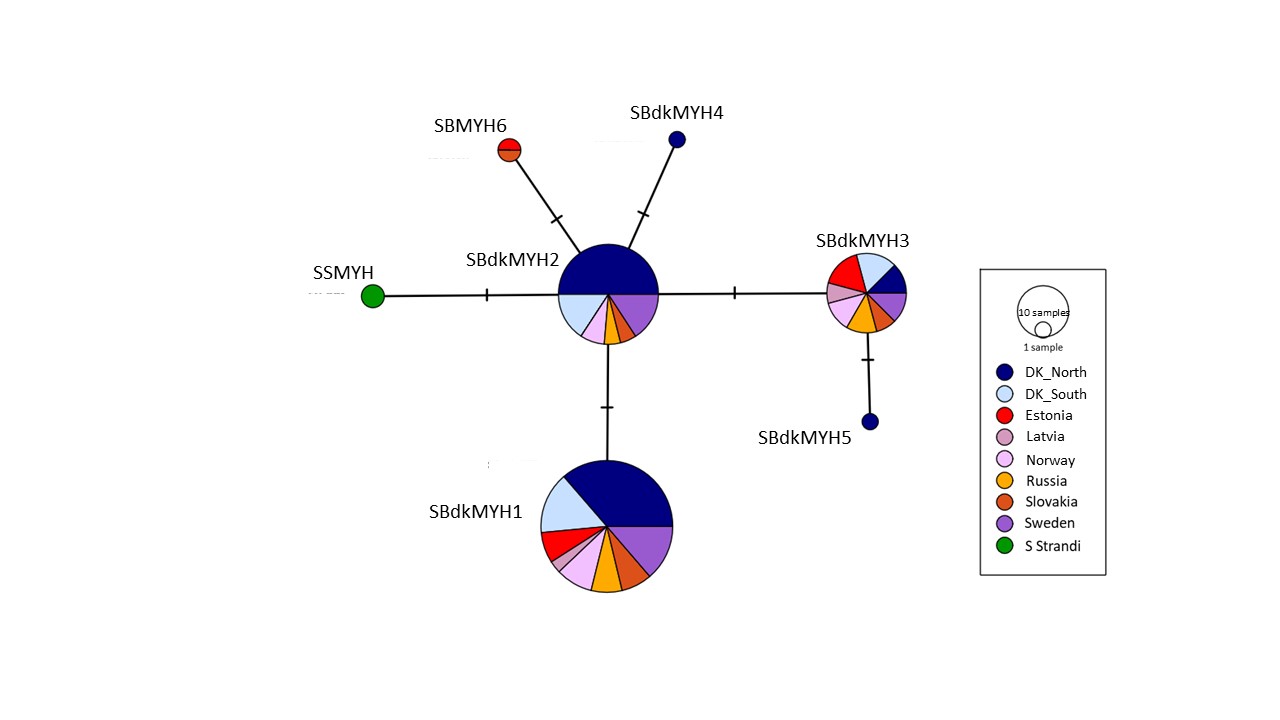


b)


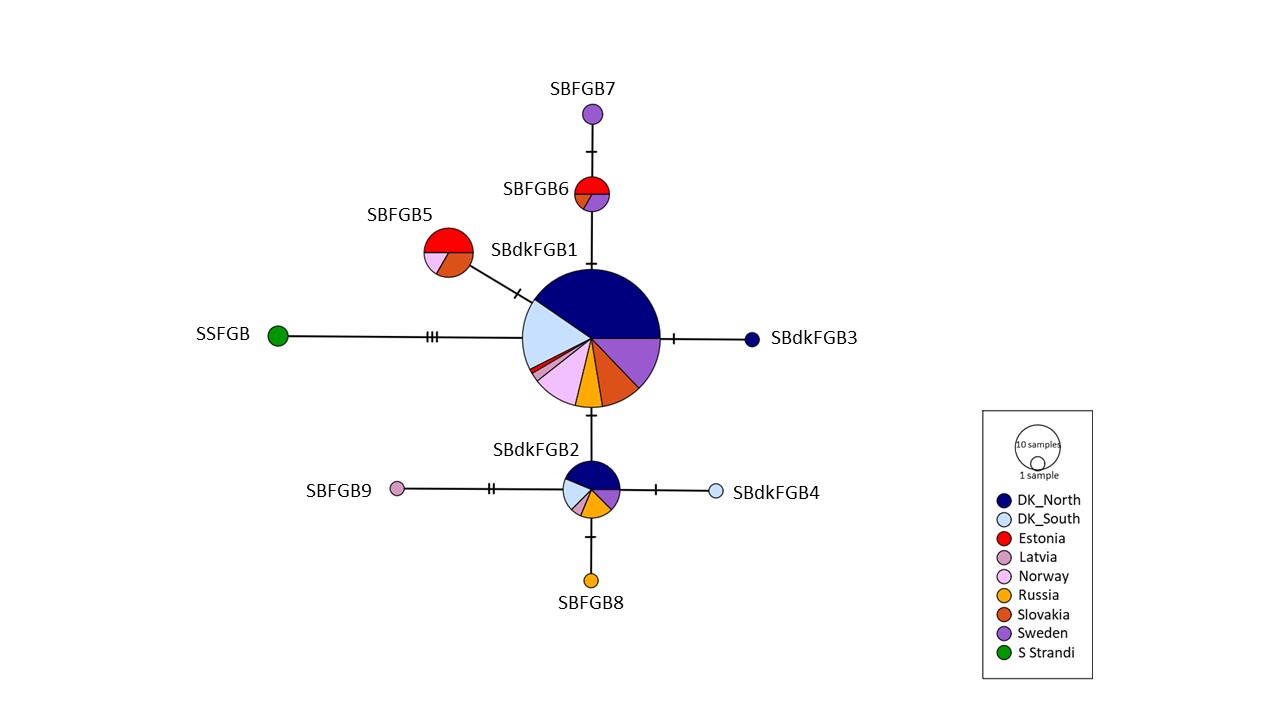


References

Leigh JW, Bryant D (2015) Data from: PopART: Full-feature software for haplotype network construction. *Methods Ecol Evol*: 1110-1116. doi: 10.1111/2041-210X.12410.

Librado P, Rozas J (2009) DnaSP v5 software for comprehensive analysis of DNA polymorphism data. *Bioinformatics* **25**: 1451-1452.

Murrell B, Wertheim JO, Moola S, Weighill T, Scheffler K, Pond SLK (2012) Detecting individual sites subject to episodic diversifying selection. *PLoS Gen* **8**: e1002764. Doi: 0.1371/journal.pgen.1002764.

Murrell B, Moola S, Mabona A, Weighill T, Sheward D, Pond SLK, Scheffler K (2013) FUBAR: A Fast, unconstrained Bayesian appRoximation for inferring selection. *Mol Biol Evol* **30**: 1196-1205. Doi: 10.1093/molbev/mst030.

Triant DA, Dewoody JA (2006) Accelerated molecular evolution in Microtus (Rodentia) as assessed via complete mitochondrial genome sequences. *Genetica* **128**: 95-108.
